# Identification and attribution of weekly periodic biases in global epidemiological time series data

**DOI:** 10.1186/s13104-025-07145-y

**Published:** 2025-02-20

**Authors:** Kit Gallagher, Richard Creswell, David Gavaghan, Ben Lambert

**Affiliations:** 1 Department of Computer Science, University of Oxford, 7 Parks Rd, Oxford, OX1 3QG UK; 2 College of Engineering, Mathematics and Physical Sciences, University of Exeter, North Park Rd, Exeter, EX4 4QF UK; 3 Department of Statistics, University of Oxford, 24 St Giles, Oxford, OX1 3LB UK; 4 Pandemic Sciences Institute, University of Oxford, Old Road Campus, Oxford, OX3 7DQ UK

**Keywords:** Time series data, Weekly oscillations, Weekend effect, COVID-19, Cholera

## Abstract

**Objective:**

COVID-19 data exhibit various biases, not least a significant weekly periodic oscillation observed in case and death data from multiple countries. There has been debate over whether this may be attributed to weekly socialising and working patterns, or is due to underlying biases in the reporting process. We investigate these periodic reporting trends in epidemics of COVID-19 and cholera, and discuss the possible origin of these oscillations.

**Results:**

We present a systematic, global characterisation of these weekly biases and identify an equivalent bias in the current Haitian cholera outbreak. By comparing published COVID-19 time series to retrospective datasets from the United Kingdom (UK), we demonstrate that the weekly trends observed in the UK may be fully explained by biases in the testing and reporting processes. These conclusions play an important role in forecasting healthcare demand and determining suitable interventions for future infectious disease outbreaks.

**Supplementary Information:**

The online version contains supplementary material available at 10.1186/s13104-025-07145-y.

## Introduction

Many time-varying epidemiological data are affected by cyclical trends on weekly or annual time scales. Weekly cycles in disease incidence or death rates can arise from a variety of factors, which may range from genuine underlying trends in the epidemiological events themselves, to mere artefacts of the observation processes involved in collecting and reporting data. For example, certain cardiac illnesses are more common on Mondays, possibly driven by factors such as alcohol consumption or employment-related stress [1–3]; while the impact of health-seeking behaviour (where individuals are more likely to access disease testing and other healthcare services on particular days of the week) is well reported across diseases from HIV [4] to influenza [5, 6]. Weekly trends in reported data may also be due to the data processing and reporting processes themselves, making the reported case numbers a biased estimate of true incidence. For example, reported mpox cases in England were lower on Fridays and Saturdays, which has been attributed to reduced test processing over the weekend [7].

A significant weekly periodic oscillation has been identified in US COVID-19 time series datasets for both case and death data [8], which has since been substantiated by observations of Huang et al. [9] on aggregated international data. Bergman et al. [8] attribute periodic oscillation in COVID-19 data from New York and Los Angeles to weekly fluctuations in case and death reporting (such as consistent under-reporting at weekends compensated by over-reporting during the week), a hypothesis supported by the analysis from Hotz et al. of case incidence data in Germany [10]. This association is disputed, however, and it has been suggested that these fluctuations reflect an underlying periodicity in the true case/death data, either due to weekly variation in inter-generational interactions [11], working patterns [12], or even an underlying circadian rhythm of the virus [13]. Oscillations in the death data are then assumed to lag oscillations in the case data by a fixed period [11], though other authors have suggested these may be a product of the ‘weekend effect’ [14], or increased stress in the clinical system [15].

To distinguish biases in the reporting process from these other effects, we may differentiate between the timing of primary and secondary events (note that this formalisation is distinct from the primary and secondary infection events common in analysis of the generation time). When considering the reporting of infections, the primary event is the infection event itself (when one individual transmits the disease to another), while the secondary event corresponds to the individual reporting a positive test for the disease. As we typically only have data for the occurrence of secondary events, any periodic variation in the secondary events may originate from periodic variation in the primary event (in this case weekly patterns in social contacts that drive infections) or biases from the secondary event (such as healthcare-seeking behaviour or reporting biases).

We may make a similar distinction in the data on deaths recorded, where there is some delay between the primary event (the death occurring) and the secondary event (the recording of the death on an epidemiological database). While death data grouped by occurrence date (the primary event) ultimately provide the most accurate representation of the pandemic, these are only available in a handful of countries/regions. Furthermore, to avoid the right truncation of deaths that have occurred but have not yet been recorded, these data are typically only available after a significant time lag to ensure that all historic events are included. Decision-making agencies therefore rely on deaths grouped by reporting date (the secondary event) [16], which may represent the best possible understanding of the pandemic at a given point in time, but are hampered by delays in reporting [17].

In this paper, we characterise these periodic reporting trends, considering both case and death data for COVID-19 globally, and discuss the possible origin of these trends. We further identify similar periodic trends in an ongoing outbreak of cholera in Haiti.

While these datasets group both cases and deaths by the date reported (hereafter referred to as ‘reporting date’), we further analyse a dataset from the United Kingdom (UK) that contains both between deaths grouped by the reporting date (i.e. the secondary event) and deaths grouped by the actual date of death, as recorded on the death certificate (i.e. the primary event). While these primary event data are not immune from reporting delays, only being updated days or even weeks after the date in question, they likely eliminate systematic, periodic biases in reporting. In post-hoc analysis, the primary event data therefore offer a unique opportunity to determine whether reporting process or genuine trends in disease events are responsible for periodic oscillations observed in datasets grouped by publication date. Identifying the origin of periodic variation in reported epidemic data is crucial for modern methods to infer epidemiological parameters [18], and underpins forecasting for modern healthcare demands such as the numbers of intensive care beds [19].

## Main text

### Methods and data

The COVID-19 data used in this report were extracted from the Johns Hopkins Database [20], up to 1st March 2023. Case rates are based on the total number of positive tests (accounting for individuals taking multiple tests), while death rates are defined as the number of deaths with a positive COVID-19 test in the past 28 days. The raw data give daily cumulative totals for both cases and deaths, which we used to generate daily case incidences and daily deaths. Any negative counts (resulting from a decrease in the cumulative total) were attributed to changes in the reporting mechanism and excluded from further analysis.

Case incidence time series of the cholera outbreak in Haiti was obtained from the PAHO/WHO Cholera dashboard up to 4th April 2023, available at: https://shiny.paho-phe.org/cholera/. This combines case reports from all 10 departments of Haiti reported by the Haiti Ministry of Public Health and Population. UK data used in the “Role of data reporting in weekly oscillation” section was extracted from the United Kingdom Health Security Agency public dashboard, using the Public Health England API service. These data are available at: https://ukhsa-dashboard.data.gov.uk/. This dataset provides two distinct records of COVID-19 deaths—the first is equivalent to the dataset provided by the Johns Hopkins database, and was available in real-time during the pandemic. This record groups death events by the date on which they were processed and added to the national database, hereafter referred to as the ‘*reporting date*’. The second record groups deaths by the true date of death, as recorded on the death certificate, which we will refer to as the ‘*event date*’.

To characterise the periodic biases observed in all datasets, we define a reporting factor $\alpha _{i}$ for each day *i*, given by the ratio between the observed value on a given day and the average value from a seven-day window centred on the given day. To determine the statistical significance, across a whole time series, of the differences between the distribution of reporting factors for each day of the week, we employ the Kruskal-Wallis H test [21]. This is a non-parametric statistical test that compares the median ranks of multiple independent groups to determine if samples come from the same distribution—in this case we compare the reporting factors grouped by day of the week. Under the null hypothesis of no variation, the test statistic has a $\chi ^{2}$ distribution.

We further compute the average reporting factor for each day of the week across the 200 countries included in the Johns Hopkins database. We reduce the dimensionality of this data through a Principal Component Analysis (PCA); this combines trends in multiple input variables (i.e. daily reporting factors) to summarize global patterns (such as weekend under-reporting) in the weekly reporting trends of COVID-19. We treat each country’s data as a row of the table, with 7 data columns representing the reporting factors for each day of the week. No further standardisation is required for this dataset, as all the input variables already vary over the range 0–7. We record the first two principle components: these are eigenvectors of the covariance matrix with the largest associated eigenvalues. As such, the sign of the PCA components is arbitrary, and so we match these to the sign of the reporting trend (i.e. positive coefficients correspond to over-reporting on the days in question). This is done by computing the inner product of each principal component with the weekly reporting trend vector for each country, identifying the 20 countries with the largest magnitude inner product (that exemplify the most extreme trends described by each principal component), and switching the sign of the principal component vector if necessary so that the majority of the largest inner products are positive. For both inner products, over 80% of the 20 countries with the largest magnitude inner products had the same sign, demonstrating the consistency in reporting trends across the dataset.

To collate these data sources and analysis, we have developed a user-friendly, open-source Python library for exploring and visualizing periodic trends in COVID-19 data. This includes notebooks for reproducing all results appearing in this paper, and is available at: https://github.com/KCGallagher/periodic-sampling.

### Results

#### Weekly reporting trends for COVID-19

**Fig. 1:**
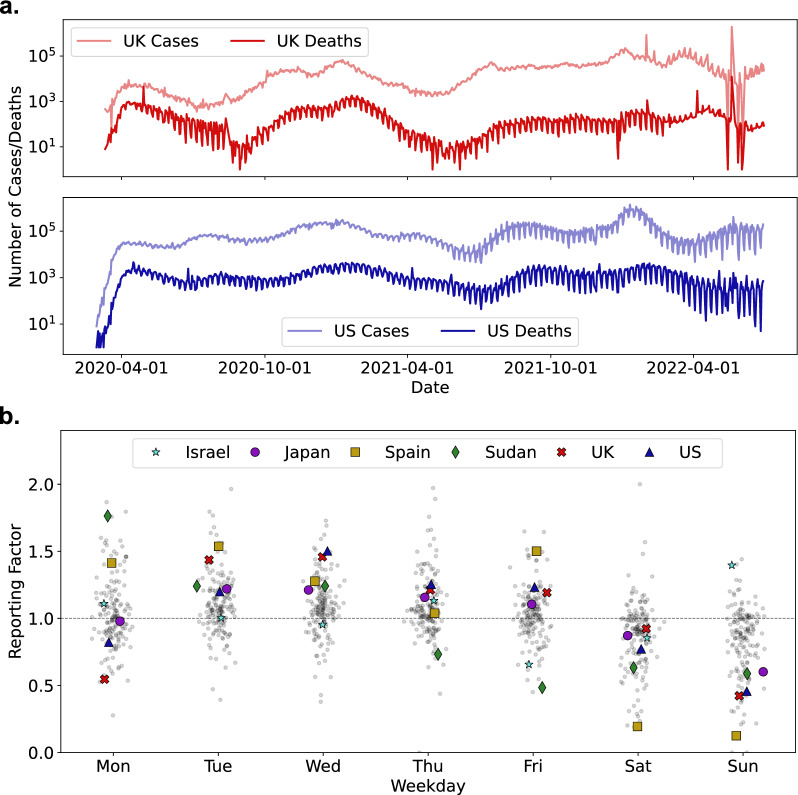
**a** Case and death time series over the COVID-19 pandemic for both the UK and US, exemplifying the weekly oscillatory pattern common among most countries. **b** Distribution of reporting factor values (grouped by day of week) for daily death statistics globally, with selected countries highlighted. (Gaussian jitter was applied to x-axis values for visualisation purposes)

Weekly reporting trends are consistently observed across countries worldwide, exemplified by the UK and US in Fig. 1a. Weekly periodicities can also be observed in the power spectral densities of these time series for both countries, plotted in Supplemental Figure S1. To demonstrate the systematic trends observed globally, Fig. 1b shows the distribution of the reporting factor for COVID-19 death data. Significant under-reporting over the weekend observed for most countries can be clearly discerned—note that for Israel the standard weekend is Friday & Saturday and under-reporting is observed on these days instead.

Table 1 gives the two primary principal components from the PCA; it is clear that the largest principal component (accounting for over a third of the variation across the dataset) corresponds to under-reporting on Saturday & Sunday (usually the weekend, though this differs across countries). More interestingly, the second principal component corresponds to the over-reporting specifically on Mondays; this principal component distinguishes between countries such as Sudan that compensate for the bulk of weekend under-reporting on Mondays alone, and countries such as Japan that compensate for weekend under-reporting by over-reporting across the working week. Both of these trends are shown in Fig. 1b, though the PCA is able to formally substantiate these visual trends.

**Table 1:** The first two principal component loadings across 200 countries illustrate the magnitude of reporting trends by day of week, with coefficients above 10 highlighted in bold

Day	PC1	PC2
Monday	3.59	**17.12**
Tuesday	8.45	0.64
Wednesday	6.83	− 4.89
Thursday	4.99	− 6.07
Friday	2.17	− 5.15
Saturday	**− 11.32**	− 4.82
Sunday	**− 14.71**	3.17
% variation	36.4	29.9

The first component demonstrates clear weekend under-reporting being compensated for during the week, while the second component suggests that over-reporting on Mondays may also be present for a set of countries

This analysis confirms a systematic, global presence of weekly periodic biases, that have previously been observed in individual countries or small-scale comparisons [8, 9, 11].

#### Weekly reporting trends for cholera

Applying these periodic analysis methods instead to daily case data from Haiti, we also observed consistent weekly trends characterised by a significant under-reporting on Sundays. Computing the Kruskal-Wallis H statistic on the reporting factor distribution between days of the week, we found significant variation in the reporting factor distribution between days of the week ($H=28,\, DF=5,\, p<0.01$).

#### Role of data reporting in weekly oscillation

**Fig. 2:**
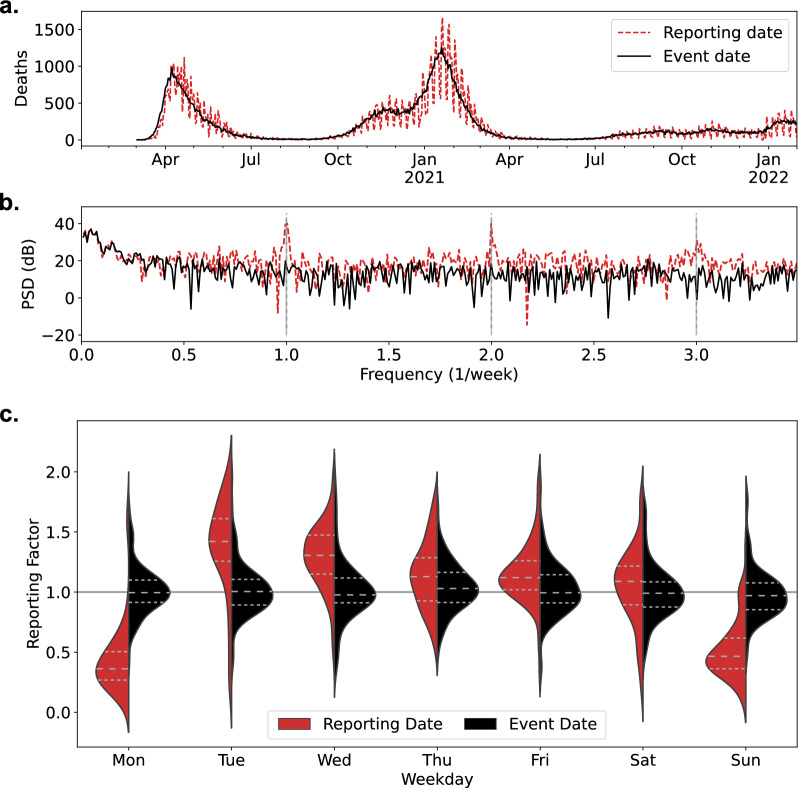
**a** Death data for the UK, grouped by both the reporting date (when the death was recorded on online reporting statistics), and the event date (as documented on the death certificate). **b** A power spectrum analysis of both time series, considering a strong periodic oscillation in the reporting data grouping, which is not observed in the event date grouping. Weekly harmonics are indicated by vertical dashed lines. **c** Distribution of reporting factors in UK death data, with interquartile ranges marked as horizontal dashed lines. A strong bias is observed in the death data grouped by reporting date, which does not occur when deaths are attributed to true event date

To inform whether the weekly oscillation observed in the “Weekly reporting trends for COVID-19” section may be attributed to solely to the reporting process (i.e. the secondary event data), or whether there is an underlying periodicity in the primary event data, we consider time series data for deaths resulting from COVID-19 in the UK. The UK death data, grouped by both the event date and reporting date, are presented in Fig. 2a.

Conducting a standard power spectrum analysis (Fig. 2b), we see a clear weekly oscillation in the time series grouped by reporting date, which appears to be eliminated in the time series grouped by event date. More formally, considering the Kruskal–Wallis H statistic for the distribution of reporting factors between days of the week in both UK death datasets, we found no evidence for weekly periodicity in the grouping by event date ($H=6,\, DF=5,\, p=0.42$), while there was strong variation in the median reporting factor for the grouping by reporting date ($H=356,\, DF=5,\, p<0.01$). The differing distribution of daily reporting factors for each dataset is shown in Fig. 2c, illustrating the weekly bias introduced by the publication process. Under-reporting is consistently observed on Sundays and Mondays, possibly corresponding to reduced death reporting over the weekend, combined with a single-day lag in data uploading to national reporting websites.

#### Impact of epidemic prevalence on reporting

To consider whether this reporting trend may be exacerbated by increased event counts (i.e. higher case or death numbers), associated with increased strain on healthcare reporting services, we considered the relationship between the reporting factor and current pandemic size. Our previous analysis has assumed that the reporting trend is a multiplicative noise term with a constant coefficient, but we consider whether this coefficient may vary with the event number over the course of the epidemic.

Conducting a linear regression on the reporting factor against the event count for each day in the UK time series data, we find a small but statistically significant, positive slope coefficient for multiple days of the week (Supplementary Table S1). However, these results may be driven by a few influential points (see Supplementary Figures S2-3), and the magnitude of the coefficients obtained corresponds to a variation in the reporting factor of < 10% over the magnitude of event counts observed during the pandemic.

### Discussion

Our work demonstrates a substantial periodic bias in COVID-19 reported case and death datasets both from the UK and across the globe, with a clear weekly frequency. Previous analysis of these datasets (including by national governments) have removed such biases with a rolling average [22, 23], however this inevitably causes a delay in the appearance of trends within the data, impacting the efficacy of government interventions. In infectious disease modelling, day-of-week effects can be incorporated into models of disease spread using, e.g., periodic functions [24] or more general day-of-week-specific delays between infection events and observed cases [25].

We use a combined dataset on primary and secondary events in UK death data to show that the weekly oscillation in this dataset can fully be explained by biases in the testing and reporting processes—primarily under-reporting at weekends. Furthermore, we show that death datasets not subject to such biases do not exhibit any weekly periodicity. Bukhari et al. [26] hypothesised that this under-reporting was a result of increased strain on health services coinciding with reduced reporting capacity at weekends. This is a possible manifestation of the ‘weekend effect’—a well-documented and debated hypothesis that clinical care standards, event reporting and general patient outcomes are lower at weekends [27–29]. This would imply that weekly oscillations in reported data would be accentuated when the healthcare services are under the most strain. Assuming epidemic prevalence is a good proxy for healthcare strain, we found a statistically significant effect, however the magnitude of this effect was insufficient to substantially affect reporting procedures at the prevalence levels during the pandemic.

We also demonstrate that similar periodic biases exist in Haitian case data from the ongoing cholera epidemic. Given the water-based transmission mechanisms of this outbreak contrast dramatically with the primarily airborne transmission of coronavirus, it is likely such weekly trends are not isolated to these two outbreaks, instead being perhaps common across many epidemiological datasets.

The World Health Organization (WHO) acknowledged that the collection and prompt publication of datasets recording occurrences of cases and deaths during the COVID-19 pandemic is crucial to the pandemic response in regions across the world [30]. In contrast, the response to the ongoing resurgence of cholera (with over 14,000 suspected cases in Haiti) has been limited by our degree of understanding of current disease dynamics. A recent (Jan 2024) WHO report [31] on the ongoing multi-country outbreak of cholera classified the global risk as ‘Very High’, reporting that strengthened surveillance and timely case management are urgently needed.

As our ability to track emerging epidemics in real-time increases due to improving temporal data resolution, it is increasingly important to understand the biases in such datasets. This allows a more informed and accurate inference of epidemiological parameters, such as delay distributions, for an ongoing pandemic [32]. These conclusions are highly relevant to healthcare providers in forecasting demand and to policymakers seeking to determine interventions for containing infectious disease outbreaks.

## Limitations

The authors only accessed death data grouped by date on the death certificate for the UK, so we can only conclude that weekly oscillations in the COVID-19 time series data are fully attributable to biases in the reporting process in this country. This result may explain similar biases we characterise in time series data reported globally, though equivalent ‘event date’ datasets would be required in each country to confirm this. Similarly, as these data are not available for the cholera epidemic we consider, we cannot confidently attribute the weekly bias we observed in cholera cases to biases in that reporting process. In particular, we highlight that (in voluntary testing settings) case data may be more affected by other weekly effects such as healthcare-seeking behaviour, in addition to biases in the underlying reporting dynamics.

We focus on time series datasets of cases and deaths. Other epidemiological time series, such as daily hospitalizations, may be expected to exhibit a wide variety of other cyclical trends, including weekly cycles, which we do not model or analyse in this study.

Furthermore, we only attribute these biases to the general reporting process and do not address the internal reporting mechanisms that contribute to widespread under-reporting over the weekend, nor to the extreme variation in reporting rates on Mondays observed in Fig. 2c and Table 1.

## Supplementary Information

Supplementary Material 1

## Data Availability

Global COVID-19 data were extracted from the Johns Hopkins Database [20], available at https://coronavirus.jhu.edu/map.html. UK-specific COVID-19 data were extracted from the United Kingdom Health Security Agency public dashboard, available at: https://ukhsa-dashboard.data.gov.uk/. Haiti Cholera data were obtained from the PAHO/WHO dashboard, available at: https://shiny.paho-phe.org/cholera/. All code for analysis and plotting are available in a user-friendly python library at: https://github.com/KCGallagher/periodic-sampling.
