# Supplementary material for: Identification and attribution of weekly periodic biases in global epidemiological time series data
Source: BMC Res Notes. 2025 Feb 20;18:78. doi: 10.1186/s13104-025-07145-y (PMC11844129; doi:10.1186/s13104-025-07145-y)
Supplement: Supplementary file 1 — Supplementary Material 1 [file 13104_2025_7145_MOESM1_ESM.pdf]

Identification and Attribution of Weekly Periodic Biases in Global Epidemiological Time Series Data

Kit Gallagher, Richard Creswell, David Gavaghan and Ben Lambert

Jan 2025

Power Spectrum Analysis

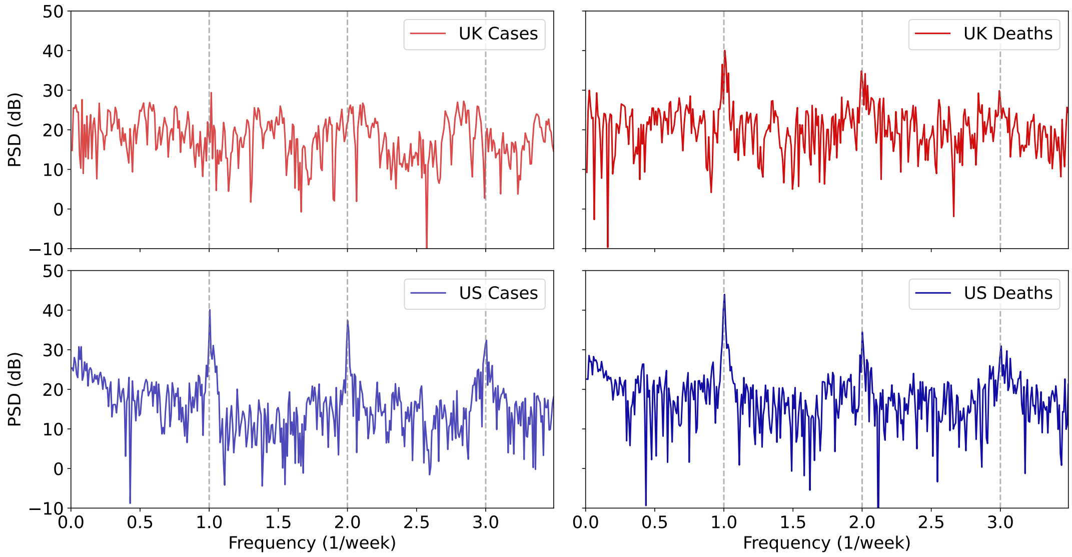

Figure S1: Power spectral densities for UK and US case and death data, with weekly harmonics marked by vertical dashed lines. Peaks in PSD at weekly harmonics demonstrate the existence of weekly periodic oscillations in the corresponding time series data.

Origin of Reporting Bias

	Monday	Tuesday	Wednesday	Thursday	Friday	Saturday	Sunday
Cases	3.87*	1.49	0.53	0.88	2.88*	-0.99	-0.97
($\times 10^{-6}$)	(0.40)	(0.71)	(0.68)	(0.62)	(0.14)	(0.65)	(0.84)
Deaths	8.51*	0.55	6.03*	0.55	4.26*	0.74	4.90*
($\times 10^{-4}$)	(1.95)	(1.43)	(0.66)	(1.25)	(0.29)	(1.14)	(1.37)

Table S1: Slope coefficients and standard error for linear regression analysis on the reporting factor against the event count in UK data. Values are scaled separately for cases and deaths, and slope coefficients that differ significantly from zero ($p < 0.05$) are marked with an asterisk.

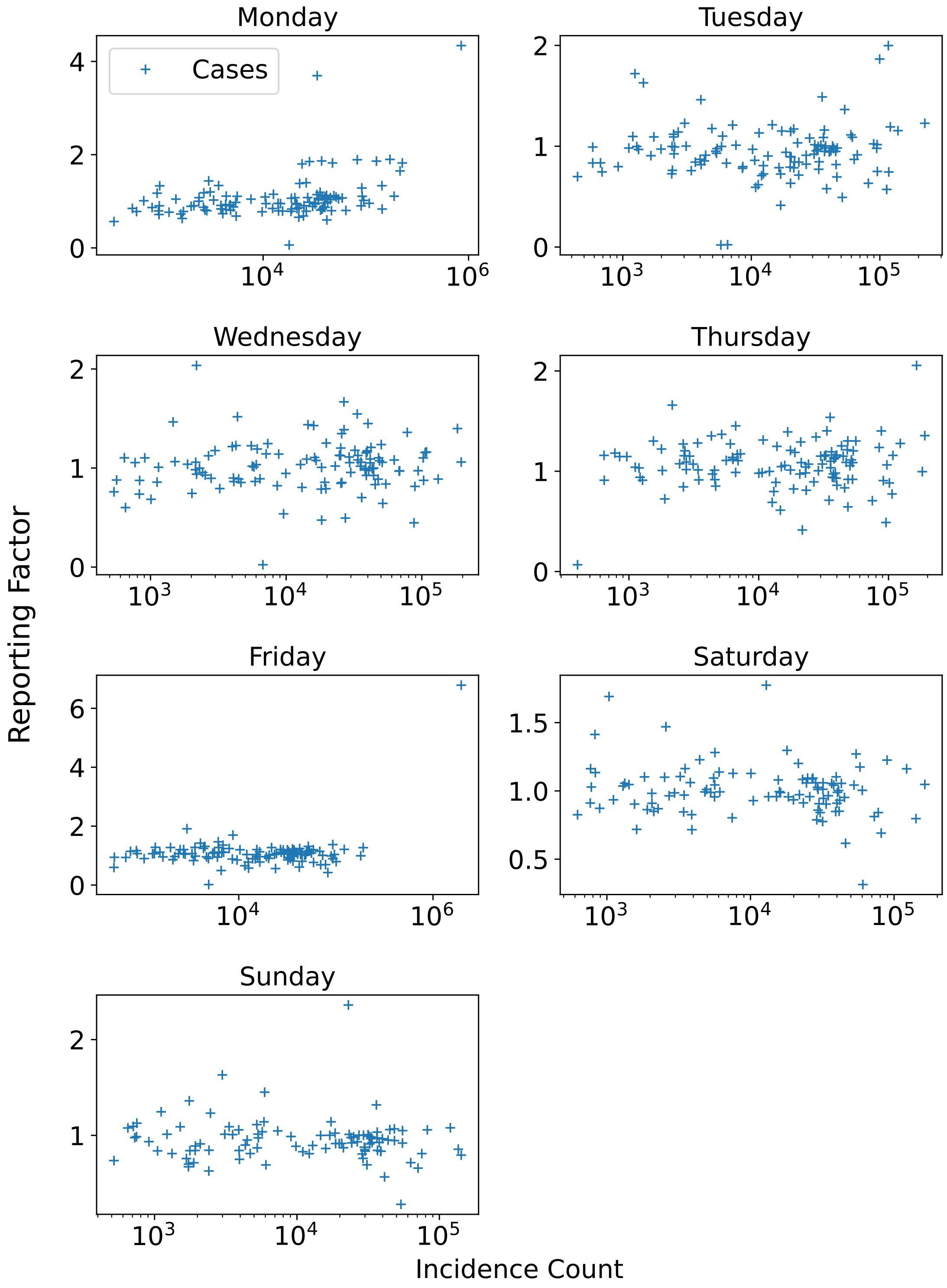

Figure S2: UK Case Data: Reporting factor against incidence count, stratified by weekday. Days with fewer than 50 cases (of which there were three in the dataset) have been excluded for visualization purposes, however they were not excluded from the regression analysis.

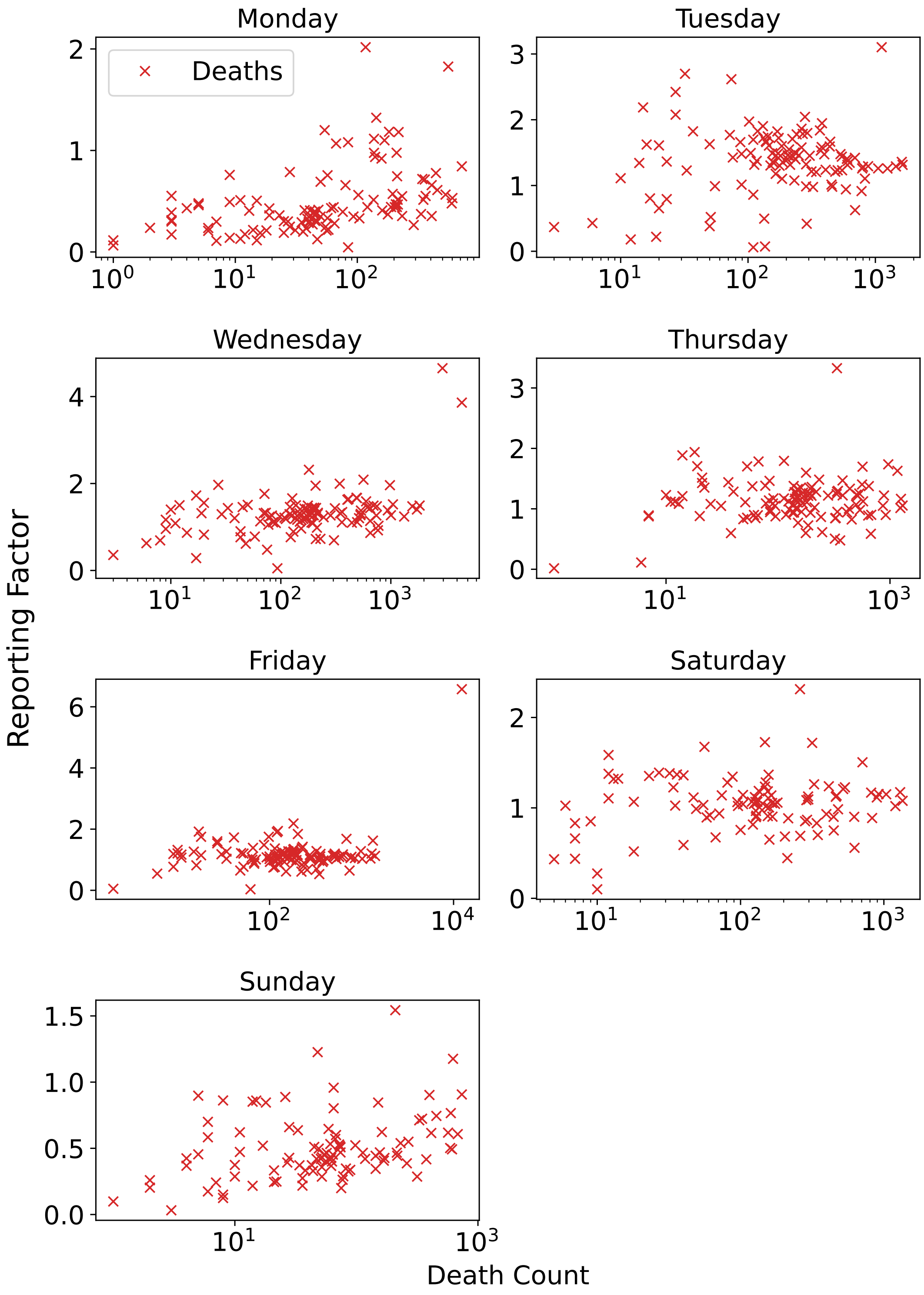

Figure S3: UK Death Data: Reporting factor against case incidence count, stratified by weekday.
